# Metabolic characteristics of solid pseudopapillary neoplasms of the pancreas: their relationships with high intensity ^18^F-FDG PET images

**DOI:** 10.18632/oncotarget.23846

**Published:** 2018-01-03

**Authors:** Minhee Park, Ho Kyoung Hwang, Mijin Yun, Woo Jung Lee, Hoguen Kim, Chang Moo Kang

**Affiliations:** ^1^ Departments of Pathology and BK21 PLUS for Medical Science, Yonsei University College of Medicine, Seoul, Korea; ^2^ Department of Hepatobiliary and Pancreatic Surgery, Department of Surgery, Yonsei University College of Medicine, Seoul, Korea; ^3^ Department of Nuclear Medicine, Yonsei University College of Medicine, Seoul, Korea; ^4^ Pancreatobiliary Cancer Clinic, Yonsei Cancer Center, Severance Hospital, Seoul, Korea

**Keywords:** solid pseudopapillary neoplasm, metabolomics, metabolism, ^18^F-FDG-PET, pancreatectomy

## Abstract

**Objective:**

We aimed to investigate the metabolic characteristics of Solid pseudopapillary neoplasms (SPNs) in relation signal intensities on ^18^F-FDG PET scans.

**Summary Background Data:**

SPNs of the pancreas commonly show high uptake of 18F-FDG. However, the metabolic characteristics underlying the high ^18^F-FDG uptake in SPNs are not well characterized.

**Materials and Methods:**

mRNA expressions for glucose metabolism were analyzed in five SPNs, five pancreatic ductal adenocarcinomas (PCAs), and paired normal pancreatic tissues. Among the proteins involved in glucose metabolism, the expressions of five proteins (GLUT1, HK1, PFKM, ENO2, and PKM2) were evaluated in 36 SPNs by immunohistochemistry. Clinical patterns of SPN on PET scans were classified according to the proportion of ^18^F-FDG uptake within the whole tumor volume (hot: ≥ 70%, mixed: 30 ≤ < 70, and defective: < 30%). PET-based parameters, including maximum standardized uptake value (SUV_max_) and metabolic tumor volume (TMV_2.5_), were evaluated.

**Results:**

Hot (*n* = 19), mixed (*n* = 5), and defective (*n* = 12) ^18^F-FDG uptake patterns were noted in the 36 patients. Radiologic tumor size and SUV_max_ differed significantly according to these patterns (ANOVA, *p* < 0.05). GLUT1, HK1, PFKM, ENO2, and PKM2 were highly expressed in SPNs at both the mRNA and protein levels. Defective type SPNs showed lower expression of HK1 (*p* = 0.014), PKM2 (*p* = 0.028), and Ki-67 (*p* = 0.070) with frequent intra-tumoral necrosis (*p* = 0.007). High Ki-67 expression (≥ 3%) was associated with high SUV_max_ in pancreatic SPNs (*p* = 0.002).

**Conclusions:**

SPN cells harbor an active molecular capacity for increased glucose metabolism. Especially, defective type SPNs were associated with low metabolic activity and related to low Ki-67 index.

## INTRODUCTION

Solid pseudopapillary neoplasm (SPN) is a very rare pathologic condition of the pancreas. It accounts for only 1–3% of all exocrine pancreatic tumors and 6–12% of all cystic tumors of the pancreas. In spite of its clinical rarity, clinical reports related with differential diagnosis have increased. In fact, a recent Korean nationwide survey of cystic neoplasms of the exocrine pancreas [1] found SPNs to rank as the third most common cystic neoplasm of the pancreas (18.3%, 195 out of 1064 patients), indicating that pancreatic SPNs may not be uncommon in Korea. Reportedly, approximately 10 to 15% of cases of SPN are malignant, although complete surgical resection of these tumors can promise long-term survival, even in cases of distant metastasis and peritoneal seeding [2–5]. Although additional genetic analysis is required to identify the exact mechanism for tumorigenesis of SPN, studies have recently found that SPNs exhibit β-catenin gene mutation [6, 7]. Diffuse cytoplasmic and nuclear localization of β-catenin is indeed found in SPN. β-catenin functions as a downstream transcriptional co-activator of the Wnt signaling pathway; therefore, the Wnt signal pathway is thought to play an important role in the tumorigenesis of SPN.

According to clinical experience and reports in the literature, [8–11]. SPNs commonly exhibit high uptake of ^18^F-FDG. However, investigations into the mechanisms for ^18^F-FDG uptake in SPNs are scarce. In 2006, Sato, et al. [12] reported poor expression of GLUT1 and moderate expression of HK2 on immunohistochemistry staining in SPN cells, suggesting that FDG accumulation might be related to tumor cell density and rich mitochondria, based on the analysis of two cases. Notwithstanding, ^18^F-FDG PET scan can reveal the metabolic and biologic activity of tumors, and it only focuses on the initial steps of glucose metabolism, such as glucose transport and hexokinase activity, not the entire pathway of glucose metabolism. Therefore, in this study, we investigated the metabolic characteristics of SPNs of the pancreas in relation signal intensities on ^18^F-FDG PET scans.

## RESULTS

### Clinical patterns of ^18^F-FDG uptake in SPNs

**Figure 1:**
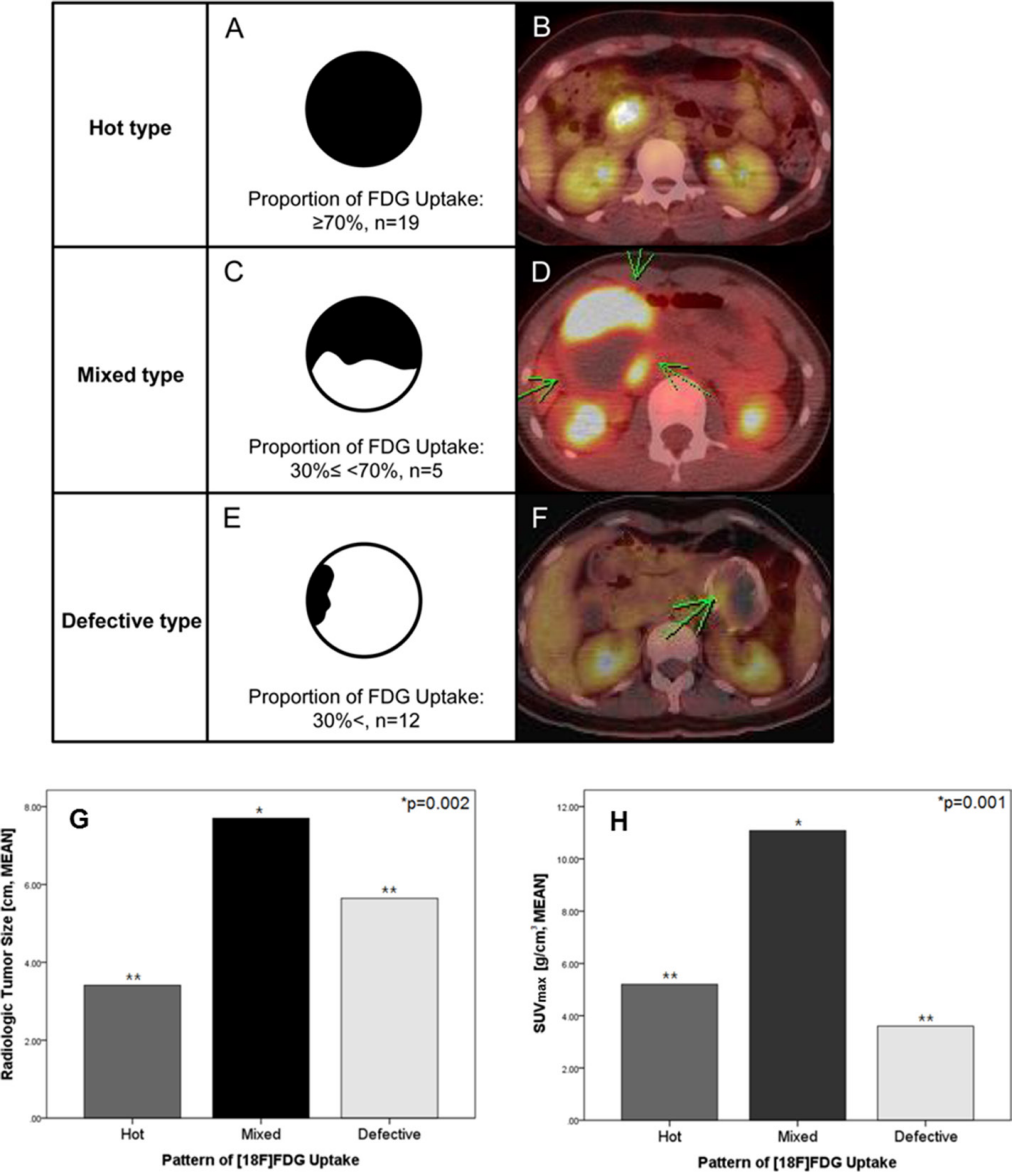
Clinical patterns of 18FDG-uptake in SPN of the pancreas Clinical patterns of ^18^F-FDG uptake in SPNs were categorized according to the proportion of ^18^F-FDG uptake over the whole tumor volume (Hot type: ≥ 70% (**A**, **B**), Mixed type: 30% ≤ < 70% (**C**, **D**), and Defective type: < 30% (**E**, **F**)). When correlating the clinical patterns of ^18^FDG-uptake with radiologic tumor size and PET-parameters, radiologic tumor size (**G**, *p* = 0.002) and SUV_max_ (**H**, *p* = 0,001) were significant different according to pattern of ^18^F-FDG uptake in SPNs of the pancreas. Mixed type of SPN was shown to be large in size with high intensity of ^18^FDG uptake. (^**^ANOVA, *p* > 0.05).

36 patients with SPNs underwent an ^18^FDG-PET scan during preoperative evaluation. 35 patients (97.2%) were female and only one was male, with an overall mean age of 34.8 ± 11.2 years. Radiologic tumor size was 4.8 ± 2.8 cm in maximum diameter. PET-related parameters were calculated in all SPNs. SUV_max_ was 5.5 ± 4.1 (g/cm^3^), and MTV_2.5_ was 31.5 ± 59.6 (cm^3^). A hot uptake pattern was identified in 19 patients (Figure 1A, and 1B, 52.8%), mixed pattern in five (Figure 1C, and 1D, 13.8%), and defective pattern in 12 patients (Figure 1E, and 1F, 33.4%).

When analyzing clinical patterns of ^18^FDG-uptake with radiologic tumor size and PET-parameters, radiologic tumor size (*p* = 0.002) and SUV_max_ (*p* = 0,001) differed significantly according to pattern of ^18^F-FDG uptake in SPNs. Mixed type SPNs were larger in size and showed higher intensities of ^18^FDG uptake (ANOVA, *p* < 0.05, Figure 1G, 1F and Supplementary Table 1).

### Gene expression profiles for glucose metabolism in SPNs

**Figure 2:**
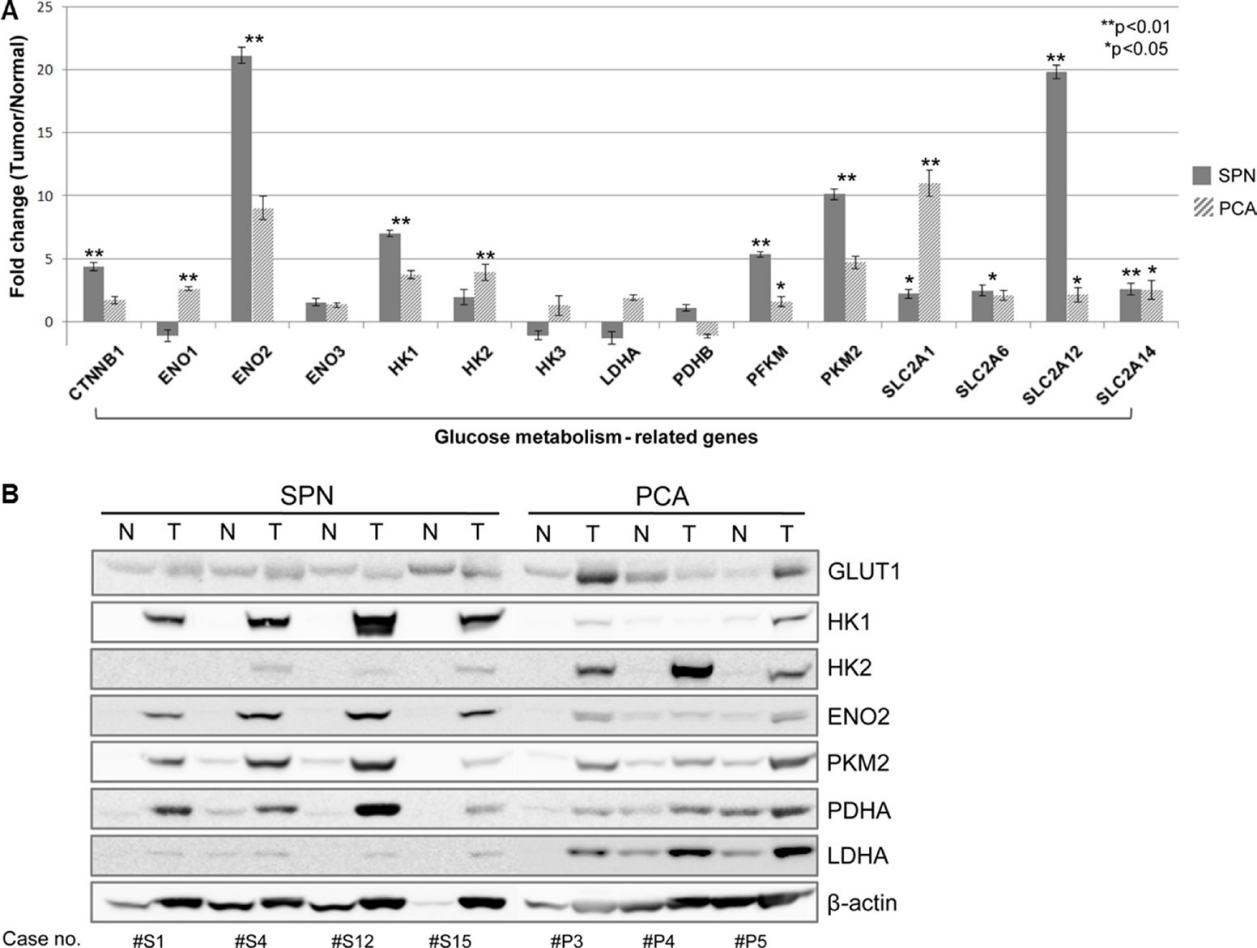
mRNA expression profiles and protein levels of genes for glucose metabolism in SPN Gene expression profiles of SPNs showed increased expression of genes involved in glucose metabolism. Note that LDHA is highly expressed in PCA comparing to normal pancreatic tissue, but it slightly decreased in SPN without statistical significance (**A**) See also Supplementary 2). Protein levels of HK1, ENO2, and PKM2 were overexpressed. Expression of HK1 and ENO2 were upregulated specifically in SPNs, compared to PCAs. Meanwhile, protein expression of GLUT1 was similar between SPNs and PCAs. The expressions of PDHB and LDHA in SPNs were similar to those in normal pancreatic tissue, although expression of LDHA was greater in PCAs (**B**). SPN, solid pseudopapillary tumor; PCA, pancreatic ductal adenocarcinoma.

We analyzed and compared the expression of genes involved in glucose metabolism and β-catenin in SPNs, normal pancreas and PCA specimens. These data were selectively obtained from our previous microarray study [13]. Typically, greater over-expression of β-catenin was noted in SPNs, compared with PCA (4.3-fold in SPN compared to normal pancreatic tissue, *p* = 0.003, and 1.6-fold in PCA, *p* > 0.05; Figure 2A and Supplementary Table 2). Expression of GLUT1 was significantly higher in PCAs than SPNs (2.2-fold in SPN, *p* < 0.05, 11.0-fold in PCA, p < 0.01). However, GLUT12 was significantly higher in SPNs than PCA (in SPN, 19.8-fold, *p* < 0.001 and in PCAs, 2.1-fold, *p* = 0.067). GLUT14 was highly expressed in both SPNs and PCAs of the pancreas, compared with normal pancreatic tissue (0.001< *p* < 0.05). Glucose transporter, GLUT6, was expressed in both SPNs and PCAs, compared to normal pancreatic tissue, with significance (0.01< *p* < 0.05) (Supplementary Table 2).

Hexokinases were also highly expressed in both SPNs and PCAs, although HK1 was strongly over-expressed in SPNs, compared with PCAs (7.0-fold in SPN, *p* < 0.01 and 3.7-fold in PCA, *p* < 0.01). Meanwhile, expression of HK2 in SPNs was similar to that in normal pancreatic tissue (1.9-fold, *p* > 0.05), and higher in PCAs (4.8-fold, *p* < 0.01). In addition, PFKM (phosphofructokinase, muscle), ENO2 (enolase-2), and PKM2 (pyruvate kinase) were also significantly overexpressed in SPNs. Together, these data suggest that SPNs have a sufficient molecular apparatus from which to active glucose metabolism (Supplementary Table 2 and Supplementary Figure 2).

Interestingly, expression of PDHB (pyruvate dehydrogenase) was similar in SPNs (1.0-fold, *p* > 0.1) and PCAs (-1.1-fold, *p* > 0.1), compared to normal pancreatic tissue. Meanwhile, LDHA (lactate dehydrogenase) was significantly overexpressed in PCAs, compared with normal pancreatic tissue (, 2.9-fold, *p* < 0.01), in contrast to SPNs in which expression of LDHA was lower than that in normal pancreatic tissue, but there were no statistical difference. (-1.1-fold, *p* > 0.1) (Supplementary Table 2 and Supplementary Figure 2).

### Protein expression for glucose metabolism in SPNs

We performed western blot analysis for the expressions of GLUT1, HK1, PFKM, ENO2, and PKM2 in the same specimens used in the microarray analysis. As shown in gene expression data, overexpression of HK1, ENO2, and PKM2 were also noted at the protein level (Figure 2B). Expression of HK1 and ENO2 were apparently unique to SPNs, compared to PCAs. Greater expression of the GLUT1 gene in SPNs was noted (2.2-fold, *p* = 0.012), compared with PCA (11-fold, *p* < 0.001, Figure 2A and Supplementary Table 2); however, GLUT1 expression at the protein level was similar between SPNs and PCAs. The expressions of PDHB and LDHA in SPNs were similar to those in normal pancreatic tissue, although expression of LDHA was greater in PCAs, as observed in the gene expression data.

### Immunohistochemistry for glucose metabolism in SPNs

Most of the glucose metabolism-related genes found in the DNA microarray analysis were confirmed in immunohistochemical analysis of resected SPNs (Supplementary Figure 1 and Table 1). GLUT1 was found to be expressed in 31 patients (86.1%, Supplementary Figure 1A). Greater expression of ENO2 was also noted compared with normal acinar cells (Supplementary Figure 1C). LDHA and PDHB expression was also observed in all patients, which seemed similar to or less than that in normal acinar cells (Supplementary Figure 1E, and 1F). However, HK1 was significantly expressed in the 26 patients (Supplementary Figure 1B), and PKM2 was clearly expressed in 20 patients (76.9%, Supplementary Figure 1D). All SPNs showed very low proliferative index in Ki-67 (Supplementary Figure 1G, and 1H). Ki-67 expression less than 3% (range, 0–5%) was found in almost all patients (94.4%), and 23 SPNs (63.8%) showed Ki-67 expression less than 1%. Immunohistochemistry for identifying glucose metabolism-related genes was also performed in 5 patients with PCAs (Supplementary Table 3). As expected, GLUT1 was expressed in all 5 patients; however, HK1 was rarely expressed and no ENO2 expression was noted in PCAs. Compared to SPNs, PKM2 and LDHA were more strongly expressed in PCAs.

**Table 1:** Immunohistochemical results of glucose metabolism-related genes in SPN

Case#	Gender	Age	Tumor Size (cm)	^18^FDG-uptake pattern	GLUT1	HK1	ENO2	PKM2	Ki-67	Necrosis
1	Female	25	5.6	Hot	+	+	+	+/-	< 1%	-
2	Female	46	5	Defective	-	++	+	+	< 1%	-
3	Female	38	3.5	Mixed	+	++	+	N/A	< 1%	+
4	Female	28	5	Defective	+	N/A	+	N/A	< 1%	+
5	Female	28	6.8	Hot	+	N/A	+	N/A	< 1%	+
6	Female	24	8.2	Hot	+	++	+	+	< 1%	-
7	Female	32	1.7	Hot	+	++	+	+	1-2%	-
8	Female	25	4.9	Defective	+	++	+	+	1–2%	-
9	Female	31	5.3	Defective	+	N/A	+	N/A	0%	+
10	Female	46	6.5	Defective	+	N/A	+	N/A	0%	+
11	Female	45	2.3	Mixed	+	++	+	+	< 1%	-
12	Female	12	10.1	Mixed	+	++	+	+	3% ≤	-
13	Female	25	12.3	Hot	+	N/A	+	N/A	1–2%	+
14	Female	35	1.3	Hot	+	++	+	+	1–2%	-
15	Female	24	5.7	Hot	+	++	+	+	< 1%	-
16	Female	62	1.5	Hot	+	++	+	+	< 1%	-
17	Female	35	1.6	Mixed	+	++	+	+	< 1%	-
18	Female	14	6.9	Hot	-	++	+	+/-	3% ≤	-
19	Female	19	3.5	Mixed	-	++	+	+	1–2%	-
20	Female	47	4.2	Hot	+	++	+	+	< 1%	-
21	Male	30	3.8	Hot	+	++	+	+	< 1%	-
22	Female	48	1.5	Defective	+	++	+	+	< 1%	-
23	Female	41	2.5	Defective	+	N/A	+	N/A	< 1%	+
24	Female	38	6.5	Hot	+	N/A	+	N/A	< 1%	+
25	Female	46	2	Defective	+	++	+	+	1–2%	-
26	Female	28	8	Defective	+	+	+	+/-	3% ≤	-
27	Female	16	7	Hot	+	N/A	+	N/A	< 1%	-
28	Female	45	3	-	-	N/A	+	+/-	< 1%	+
29	Female	43	5.2	Defective	-	N/A	+	+/-	< 1%	+
30	Female	22	8.5	Hot	+	+	+	+	1–2%	-
31	Female	40	8.3	Hot	+	++	+	+	1–2%	-
32	Female	35	1.6	Hot	+	++	+	+	< 1%	-
33	Female	38	2	Hot	+	++	+	N/A	1–2%	-
34	Female	40	2.7	Hot	+	+	+	N/A	< 1%	-
35	Female	42	1.5	Hot	+	++	+	+	1–2%	-
36	Female	23	5	Hot	+	++	+	+/-	< 1%	-

N/A; not available due to necrosis, immunohistochemistry score (-; negative, +/-; weak, +; moderate, ++; strong)

### Correlation between PET-based parameters and glucose metabolism-related gene expressions in SPN

**Figure 3:**
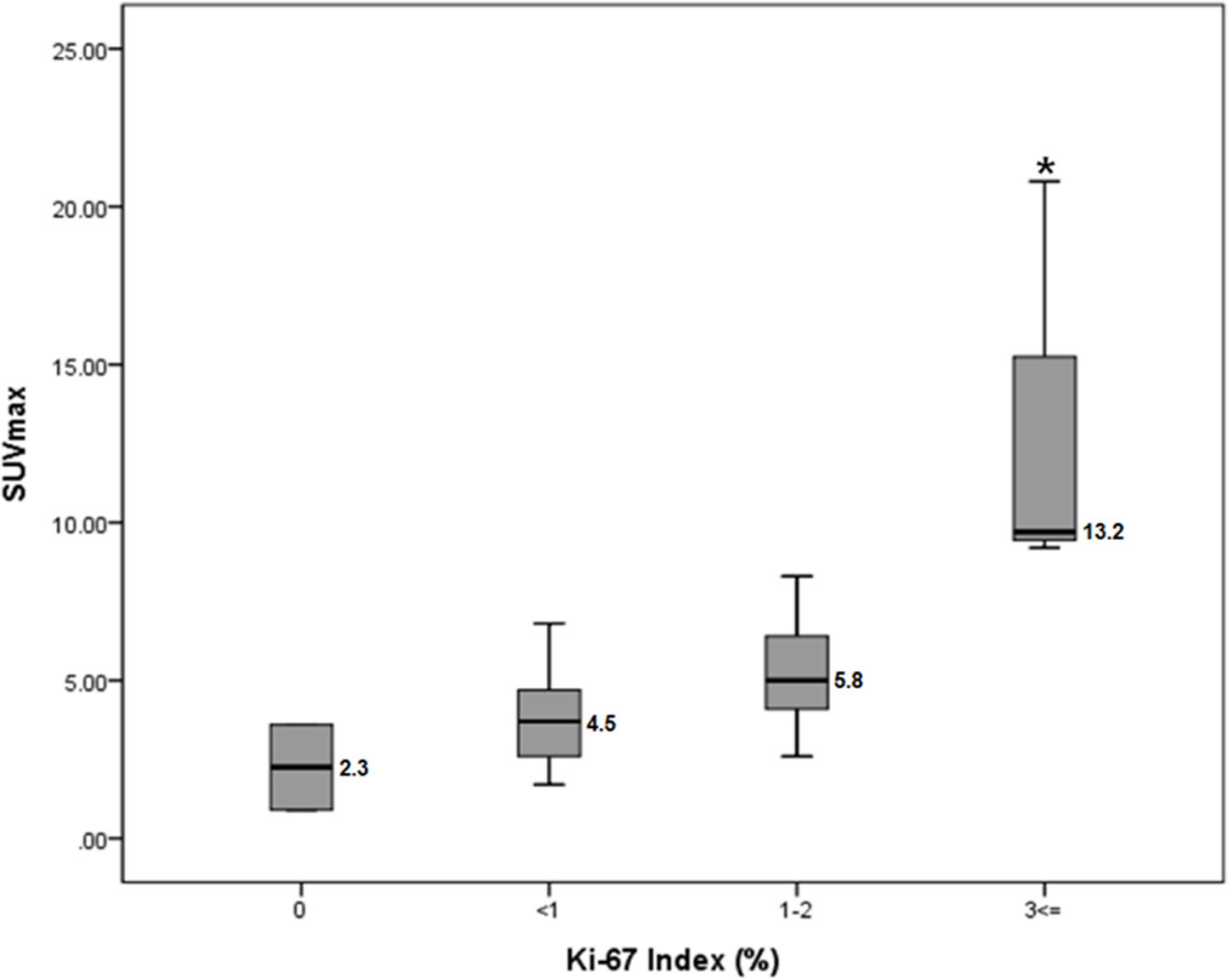
SUV_max_ values according to Ki-67 Index SPNs with high ^18^F-FDG intensity (SUV_max_) showed higher Ki-67 index (ANOVA). ^*^p = 0.002, a number beside the box shows the mean value of SUV_max_

GLUT1 expression was not associated with ^18^FDG-uptake intensity in SPNs. There was no difference in GLUT1 expression according to pattern of ^18^FDG-uptake (*p* = 0.646, Supplementary Table 4). However, expression of HK1 (*p* = 0.014) and PKM2 (*p* = 0.028) were found differ according to pattern of ^18^FDG-uptake. Expression of HK1 and PKM2 decreased in defective pattern, comparing with hot and mixed patterns (Supplementary Figure 3A, and 3B). Intra-tumoral necrosis was significantly associated with defective type SPNs (*p* = 0.007, Supplementary Figure 3C, and Supplementary Table 4). In addition, there was a significant association between intra-tumoral necrosis and Ki-67 index (*p* = 0.017, Table 2), indirectly suggesting SPNs with intra-tumoral necrosis are related with a lower Ki-67 index. Therefore, defective type had a tendency to show lower proliferation power, compared with hot and mixed SPNs (*p* = 0.07, Supplementary Figure 3D). Also, SPNs with high ^18^F-FDG intensity (SUV_max_) showed higher Ki-67 index (ANOVA, *p* = 0.002, Figure 3).

**Table 2:** Correlation between intratumoral necrosis and Ki-67 index

		Necrosis		*P*-value
		Negative	Positive	
**Ki-67 (%)**	0	0	2	0.017
	< 1	14	7	
	1–2	9	1	
	3 ≤	3	0	

### Correlation between expression of glucose metabolism-related genes, patterns of FDG uptake, and microscopic malignant feature of SPNs

Seven (19.4%) out of 36 patients were found to have microscopic malignant features in resected SPNs. Microscopic malignant features included capsule invasion, perineural invasion, vascular invasion, infiltrative to pancreatic tissue, and peripancreatic tissue invasion. PET-based parameters (SUV_max_ and MTV_2.5_) were similar between SPN groups with microscopic benign and microscopic malignant features (*p* > 0.05, Table 3). There were no relationships between expression of GLUT1, HK1, and PKM2, as well as Ki-67 index, and microscopic malignant features (*p* > 0.05). However, defective type SPNs tended to be associated with benign-looking SPNs, compared with hot and mixed type SPNs, although this difference did not reach statistical significance (*p* = 0.070, Table 4).

**Table 3:** Correlation between PET-based parameters and microscopic malignant features of SPNs

	Microscopic malignant features		*P*-value
	Absent (*N* = 29)	Present (*N* = 7)	
SUV_max_	5.1 ± 4.0	6.9 ± 4.5	0.299
MTV_2.5_	23.3 ± 47.2	65.3 ± 92.8	0.284
Tumor size	4.9 ± 3.2	5.1 ± 2.9	0.828

**Table 4:** Correlation between staining intensity of glucose metabolism-related genes and microscopic malignant features of SPNs

		Microscopic malignant features		*P*-value
		Absent (*N* = 29)	Present (*N* = 7)	
**GLUT1**	-	4	1	1.000
	+	25	6	
HK1^*^	+	3	1	1.000
	++	17	5	
PKM2^*^	+/-	6	0	0.280
	+	14	6	
**Necrosis**	-	20	6	0.645
	+	9	1	
**Ki-67**	0	2	0	0.781
	< 1	15	6	
	1–2	10	-	
	3 ≤	2	1	
**Clinical pattern ^18^FDG-uptake** **Defective**	Hot+Mixed	17	7	0.070
	12	0		

^*^ 10 cases were not included due to poor staining.

## DISCUSSION

To date, SPN characteristics on ^18^F-FDG PET or PET/CT have rarely been reported. According to the literature, the intensity of ^18^FDG-uptake in SPNs varies widely. In some reports, [14, 15] SPNs with mild or no ^18^F-FDG uptake have been presented, while others have described SPNs with intense ^18^F-FGD uptake [8, 9, 12, 16, 17]. With accumulating clinical experience in SPN, application of PET scans to SPNs of the pancreas has expanded to testing its capacity to differentiate SPNs from other malignant tumors, such as pancreatic cancer or neuroendocrine tumor, [10, 11, 18, 19] in addition to its clinical usefulness in staging and treatment planning [16, 17]. Although several reports of high ^18^F-FDG uptake in SPN have been published, research on the metabolic mechanisms of SPNs is rarely reported. It is thought that any tumor will cause the alterations of the glucose metabolism, then glucose metabolism should not be the specific for SPNs. However, there was no study to investigate glucose metabolic alteration in SPNs to confirm this assumption. The exact mechanisms involved in glucose uptake by SPNs are still unknown. Therefore, we primarily tried to overview the landscape of altered glucose metabolism in resected SPNs of the pancreas, especially comparing with pancreatic cancer.

In present study, based on our previous research of characterizing gene expression profiles for SPNs, [13]. we investigated the molecular mechanisms of ^18^F-FDG uptake in SPNs. To the best of our knowledge, the present study is the first to report on a molecular mechanism of glucose metabolism in SPNs. According to our data, SPNs show a distinct molecular apparatus for glucose uptake and for glycolysis to form pyruvate. We found that GLUT1, GLUT6, GLUT12, and GLUT14 are highly expressed in SPNs. GLUT1, known as a principal glucose transporter in tumors, was expressed even at the protein level. In addition, enzymes involved in glycolysis, such as HK1, ENO2, and PKM2, were also overexpressed, and this was confirmed in both western blot and immunohistochemistry experiments. These molecular profiles strongly suggest that neoplastic cells of SPN possess an increased capacity for glucose metabolism, which is observable in the appearance of SPNs on ^18^F-FDG -PET scans.

This study also attempted to categorize clinical patterns of ^18^F-FDG uptake in SPNs. We previously classified patterns of ^18^F-FDG uptake in SPNs into five categories [20]. In addition, we also tried to categorize SPNs according to different ^18^F-FDG-uptake proportion such as, < 10%, 10%–30%, 30–50%, 50–70%. 70–90%, and 90% <, and the similar relationship with the present results were founded (data not shown). These classification system looks very specific, but it was found that these categories were complex and confused to apply in clinical practice. So, we slightly modified previous five categories simply into Hot uptake (previously belong to type I and type II), Mixed (previously belong to type III), and Defective type (previously belong to type IV and V) in this study based on the proportions of ^18^F-FDG uptake within the whole tumor.

In the present study, we found that intra-tumoral necrosis was found to be associated with low Ki-67 index (Table 2), and defective type SPNs were related to intra-tumoral necrosis and a lower Ki-67 index (Supplementary Figure 3C, and 3D). These observations indirectly suggest that defective type SPNs may be less likely to progress. Defective type SPNs, resulting from intra-tumoral hemorrhagic necrosis due to weakened cell-to-cell adhesion, [21] can cause chronic hypoxia and ischemic necrosis of viable solid portions of the tumor. If the tumors are not clinically detected, even at this stage, and/or not treated immediately, a totally necrotic tumor of the pancreas might be found. Recently, we proposed that marginally calcified, totally necrotic pancreatic tumors might comprise a subset of SPNs with near total necrosis [22] (perhaps defective type SPN), and the present observation may support this hypothesis. It is also interesting to note that the clinical patterns of ^18^F-FDG uptake in SPNs apparently represent the metabolic activity of SPN. Expression of HK1 and PKM2 were closely correlated with patterns of ^18^F-FDG uptake (Supplementary Figure 3A, 3B and Supplementary Table 4). More frequent expression of HK1 and PKM2 were noted in hot and mixed pattern, compared to the defective pattern. Pyruvate kinase is the last rate-limiting enzyme in glycolysis, and catalyzes the conversion of phosphoenolpyruvate and ADP into pyruvate and ATP. PKM2 is known to be expressed predominantly in tumor cells, and is important for cancer metabolism and tumor growth. A previous study showed that PKM2 expression is involved in early tumorigenesis [23]. and that increases in PKM2 levels are correlated with tumor size and stage [24]. HK catalyzes the conversion of glucose to glucose-6-phosphate, the first and rate-limiting step in the glycolytic pathway. Usually, HK2 is regarded as a principle enzyme in cancer metabolism; however, our study suggests that HK1 is the main enzyme converting glucose to glucose-6-phosphate in SPNs. Nevertheless, the exact role of HK1 and PKM2 in the tumorigenesis of SPN remains to be investigated further (Supplementary Figure 2).

We also found that SPNs with high Ki-67 index are related with high SUV_max_ (Figure 7). Tumor size and PET-related parameters, such as SUV_max_, were larger and higher in mixed type SPNs (Figure 2 and Supplementary Table 1), suggesting mixed type SPNs may be a biologically active tumor with higher Ki-67 index. Due to the limited number of mixed type SPNs in our data set (*n* = 5 patients), it would have been difficult for us to definitively examine this potential relationship; however, this observation is thought to be very important, because Ki-67 index and tumor proliferation have been previously reported to associated with aggressive biological behavior of SPNs [25–27]. Recently, Yu, et al. [28] also demonstrated that positive immunoreactivity for Ki-67 may predict the malignant potential and poor outcomes of SPN. Nakagohri, et al. [14] reported that most SPNs (5 out of 6 tumors) show strong accumulation of FDG on PET scans, and showed that SPNs with high FDG uptake were related to microscopic venous and perineural invasion. Conversely, the tumors without intense FDG uptake had neither microscopic venous invasion nor nerve invasion, suggesting a potential relationship between SUV and histological malignancy. In our data, we observed no relationship between malignant microscopic features in resected SPNs and GLUT1, HK1, intratumoral necrosis, and Ki-67 index. However, patterns of ^18^F-FDG uptake in SPNs were found to be related to microscopic malignant features of SPN with marginal significance (*p* = 0.070, Table 4).

**Figure 4:**
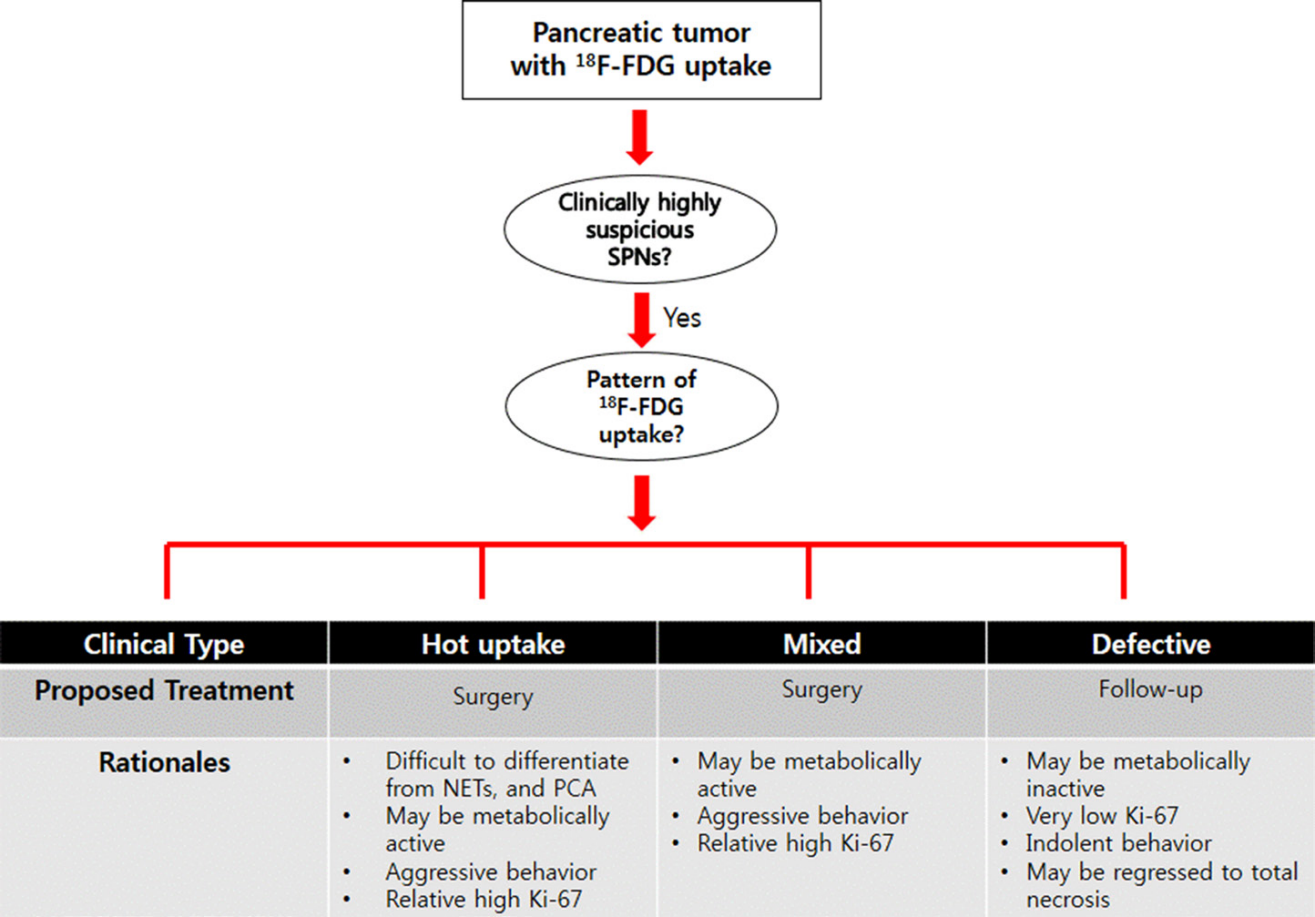
Proposing surgical strategy for SPTs of the pancreas based on the pattern of ^18^F-FDG uptake Surgical decision-making can be specified according to clinical pattern of ^18^F-FDG uptake in patients with SPN.

Therefore, clinically, it can be recommended that SPT with high SUV_max_ (for example, hot uptake and mixed type) should be aggressively treated from the metabolomics point of view, because PET-based parameter, especially high SUV_max_ was related to increased Ki-67 index. In addition, small hot-uptake type of SPT should be considered for resection because it is difficult to differentiate from other malignant tumors of the pancreas, such as pancreatic cancer, and neuroendocrine tumor, when the tumor did not show typical radiologic characteristics of SPNs of the pancreas [11, 18]. On the other hand, defective type of SPNs was associated with low expression of HK1/PKM2, intratumoral necrosis, and tended to have lower proliferation with low metabolic capacity (Figure 1H, and Figure 3), suggesting this metabolic type of SPNs might be less progressive and would follow indolent clinical course. Considering most patients with SPNs are young female patients with active social activity, surgery for SPTs with defective type of ^18^F-FDG uptake could be reserved according to patient’s social activity, and physical conditions, because it cannot be denied that pancreatectomy is related to high rate of postoperative morbidity with potential mortality in spite of improved perioperative management [29] (Figure 4).

Based on the current observations, further investigation of the metabolic differences between indolent type SPNs and those with clinically aggressive behavior could prove valuable. What makes indolent SPNs change to take on an aggressive biologic behavior? What differences exist between types of SPN from the view point of metabolism? Why should SPNs show increased glucose metabolism in spite of low proliferation? Is there any specific metabolomics pathway for SPNs? Future basic and clinical research should provide answers to this surgical enigma.

## MATERIALS AND METHODS

### Patient selection and clinical data

The medical records of 36 patients with SPN who underwent preoperative ^18^F-FDG PET/PET-CT scans and a pancreatectomy were retrospectively reviewed. The clinicopathologic characteristics of the patients, including age, gender, radiologic tumor size, and microscopic malignant features, were investigated. SPN tissue samples from 36 patients were obtained by surgical resection. Among them, five fresh tissues that consisted of more than 70% tumor cells without previous adjuvant chemo- or radiotherapy were selected for analysis of gene expression profiles. The specimens were obtained from the archives of the Department of Pathology, Yonsei University, Seoul, Korea and from the Liver Cancer Specimen Bank of the National Research Resource Bank Program of the Korea Science and Engineering Foundation under the Ministry of Science and Technology. Authorization for the use of these tissues for research purposes was obtained from the Institutional Review Board of Yonsei University of College of Medicine.

### ^18^F-FDP PET/ PET-CT protocol

All ^18^F-FDG PET/PET-CT scans were performed with a dedicated PET/CT scanner (Discovery STe, GE Healthcare; or Biograph TruePoint 40, Siemens Healthcare). All patients fasted for at least 6 h prior to the PET/CT scan. A dose of approximately 5.5 MBq/kg of ^18^F-FDG was intravenously injected 60 min before imaging. First, CT scans were performed at 30 mA and 130 kVp with the Discovery STe scanner or at 36 mA and 120 kVp with the Biograph TruePoint scanner without contrast-enhancement. After the CT scan was complete, a PET scan was performed from the neck to the proximal thigh, with an acquisition time of 3 min per bed position in a 3D mode. PET images were reconstructed using ordered subset expectation maximization with attenuation correction.

### Image evaluation and PET-based parameters

^18^F-FDG PET/CT images were reviewed by two nuclear medicine physicians (Yun M, and Cho A) using an Advantage Workstation 4.4 (GE Medical Systems). Maximum standardized uptake value (SUV_max_) and metabolic tumor volume (TMV_2.5_) on PET images were measured using the volume viewer software. Each tumor was examined with a spherical-shaped volume of interest (VOI) that included the entire lesion in the axial, sagittal, and coronal planes. By using CT images, ^18^F-FDG uptake by normal organs, such as the bowel, stomach, and liver, was excluded from the VOI. The SUV_max_ of the VOI was calculated as (decay-corrected activity/tissue volume)/(injected dose/body weight). MTV_2.5_ was defined as the total tumor volume with an SUV ≥ 2.5. In patients with a SUV_max_ of < 2.5, MTV_2.5_ was not measured. In addition, clinical patterns of ^18^F-FDG uptake in SPNs were categorized according to the proportion of ^18^F-FDG uptake over the whole tumor volume (hot: ≥ 70%, mixed: 30% ≤ 70%, and defective: < 30%, Figure 1).

### mRNA gene expression data preparation and statistical analysis

Raw data were extracted using the software provided by Illumina Genome Studio v2011.1 (Gene Expression Module v1.9.0). Expression intensities were normalized using quantile normalization techniques. Using the normalized intensities, differentially expressed genes (DEGs) between non-neoplastic pancreatic tissue and pancreatic tumors (SPN, or PCA) were determined using a previously reported integrated statistical method [13]. We selected the expression of genes involved in glucose metabolism in five SPNs, and compared their results to those of normal pancreas and PCA specimens.

### Western blot

Whole lysates from tissue specimens were prepared using passive lysis buffer (Promega). Protein samples were separated by SDS-PAGE and transferred to nitrocellulose membranes. Blots were blocked with Tris-buffered saline and Tween 20 containing 5% skim milk, and incubated overnight at 4°C with primary antibodies against GLUT1 (Alpha Diagnostic), HK1, PKM2, LDHA, PDHA (Cell Signaling) and ENO2 (antibodies-online). After washing, the membranes were incubated with horseradish peroxidase-conjugated secondary antibody (Santa Cruz Biotechnology) for 1 h at room temperature, washed, and developed with luminol reagent (Santa Cruz Biotechnology).

### Immunohistochemistry

Healthy, available paraffin-embedded tissue blocks for the 36 patients were cut into 4-μm sections. Immunohistochemical analysis was performed using a Ventana XT automated stainer (Ventana Corporation) with antibodies against GLUT1 (Alpha Diagnostic), HK1, PKM2, LDHA, PDHA (Cell Signaling), ENO2 (antibodies-online), and Ki-67 (Dako). Immunohistochemical results were scored according to staining intensities as follows: -, no staining; +/-, weak staining (faint protein expression); +, moderate staining (definite protein staining in ≤ 30% of tumor cells); or ++, strong staining (definite protein expression in > 30% of tumor cells).

### Statistical analysis

Continuous variables are expressed as mean ± standard deviation and categorical variables as frequency (%). ANOVA and Student’s *t*-test were used for comparative analysis, while chi-square (Fisher’s exact test, or linear-to-linear association if necessary) was used for analyzing clinical patterns of FDG uptake and immunohistochemical grades for detecting glucose metabolism-related gene expression. Statistical analyses were performed using SPSS software, version 20.0 for Windows (SPSS Inc.). *P*-values < 0.05 were considered statistically significant.

## SUPPLEMENTARY MATERIALS FIGURES AND TABLES



## Abbreviations

SPN: Solid pseudopapillary neoplasm
Wnt: Wingless-related integration site
PCA: Pancreatic ductal adenocarcinomas
GLUT1: Glucose transporter 1
HK2: Hexokinase2
^18^F-FDG PET: Fluorodeoxyglucose positron emission tomography
CT: Computed tomography
SUV_max_: Maximum standardized uptake value
TMV: Metabolic tumor volume
PKM2: Pyruvate kinase muscle isozyme 2
LDHA: Lactate dehydrogenase A
PDHA: Pyruvate dehydrogenase E1 component subunit alpha
ENO2: enolase 2
