# Supplementary material for: Metabolic characteristics of solid pseudopapillary neoplasms of the pancreas: their relationships with high intensity 18F-FDG PET images
Source: Oncotarget. 2018 Jan 3;9(15):12009–19. doi: 10.18632/oncotarget.23846 (PMC5844725; doi:10.18632/oncotarget.23846)
Supplement: Supplementary file 1 [file oncotarget-09-12009-s001.pdf]

Metabolic characteristics of solid pseudopapillary neoplasms of the pancreas: their relationships with high intensity ^{18}F-FDG PET images

SUPPLEMENTARY MATERIALS

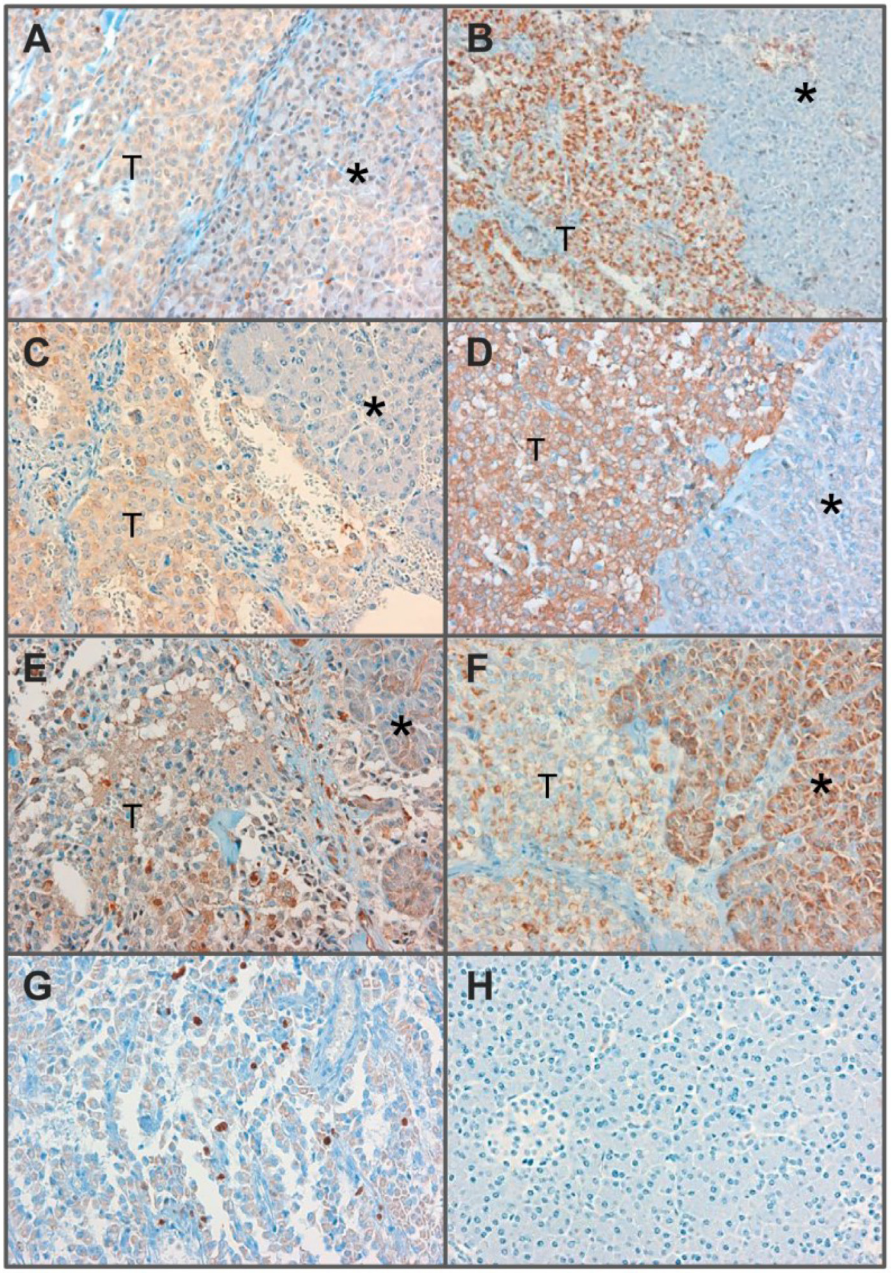

Supplementary Figure 1: Immunohistochemical analysis of glucose metabolism related genes. Expression of GLUT1 (**A**), HK1 (**B**), ENO2 (**C**), PKM2 (**D**), LDHA (**E**), PDHD (**F**), and Ki-67 in SPN (**G**), and Ki-67 in normal acinar cells (**H**). Note that * indicates area of normal acinar cells, and T indicates tumor area of SPN.

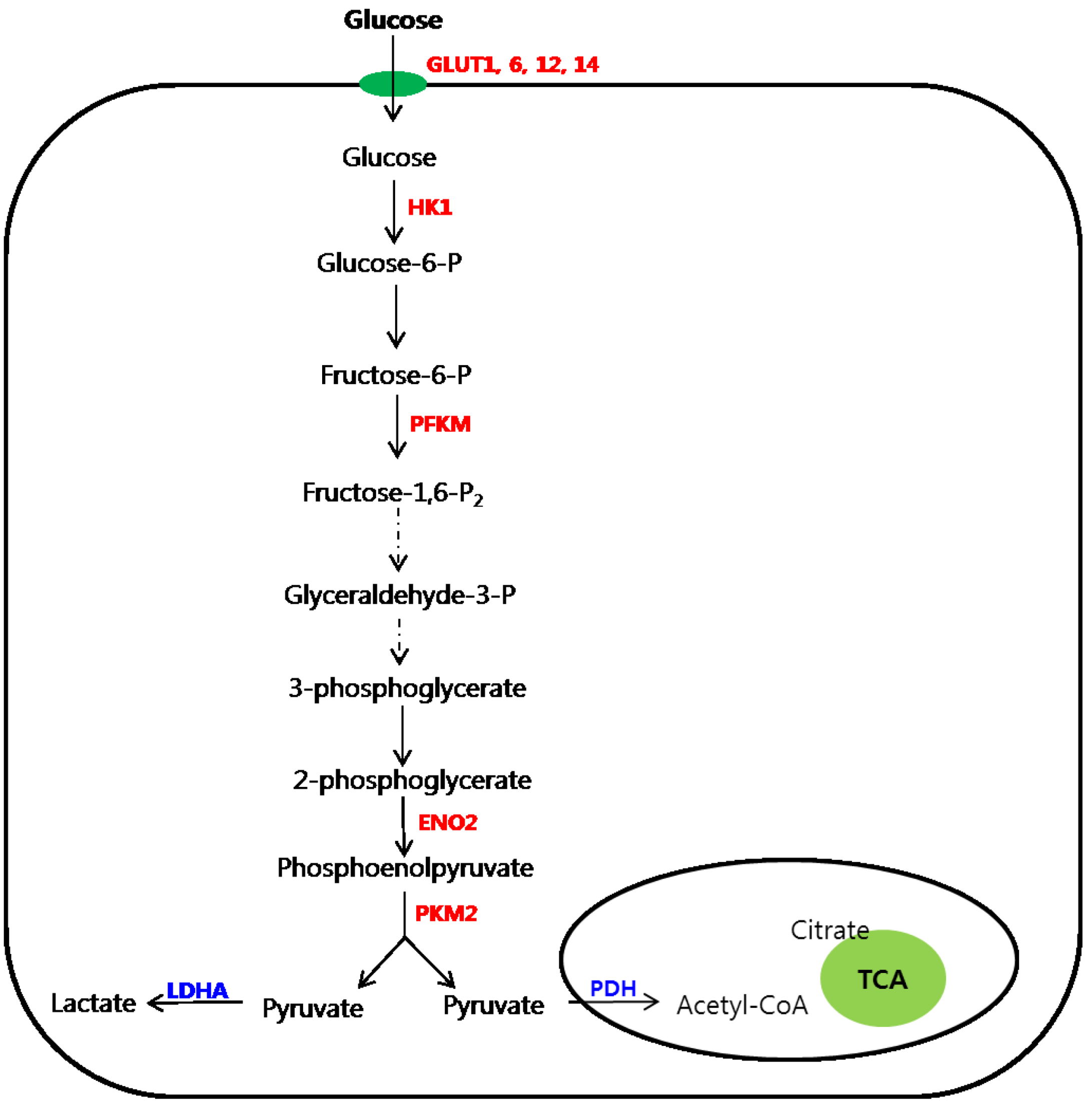

Supplementary Figure 2: Schematic summary of gene expression profiles for glucose metabolism in SPN of the pancreas. GLUT1, GLUT6, GLUT12, and GLUT14 were highly expressed in SPN compared to normal pancreatic tissue. HK1 was strongly overexpressed in SPN of the pancreas compared with PCA. PFKM (phosphofructokinase, muscle), ENO2 (enolase-2), and PKM2 (pyruvate kinase) were also significantly overexpressed in SPN of the pancreas. However, unlike pancreatic cancer, the expression of PDHB (pyruvate dehydrogenase beta) and LDHA (lactate dehydrogenase A) in SPN were similar in normal pancreatic tissue.

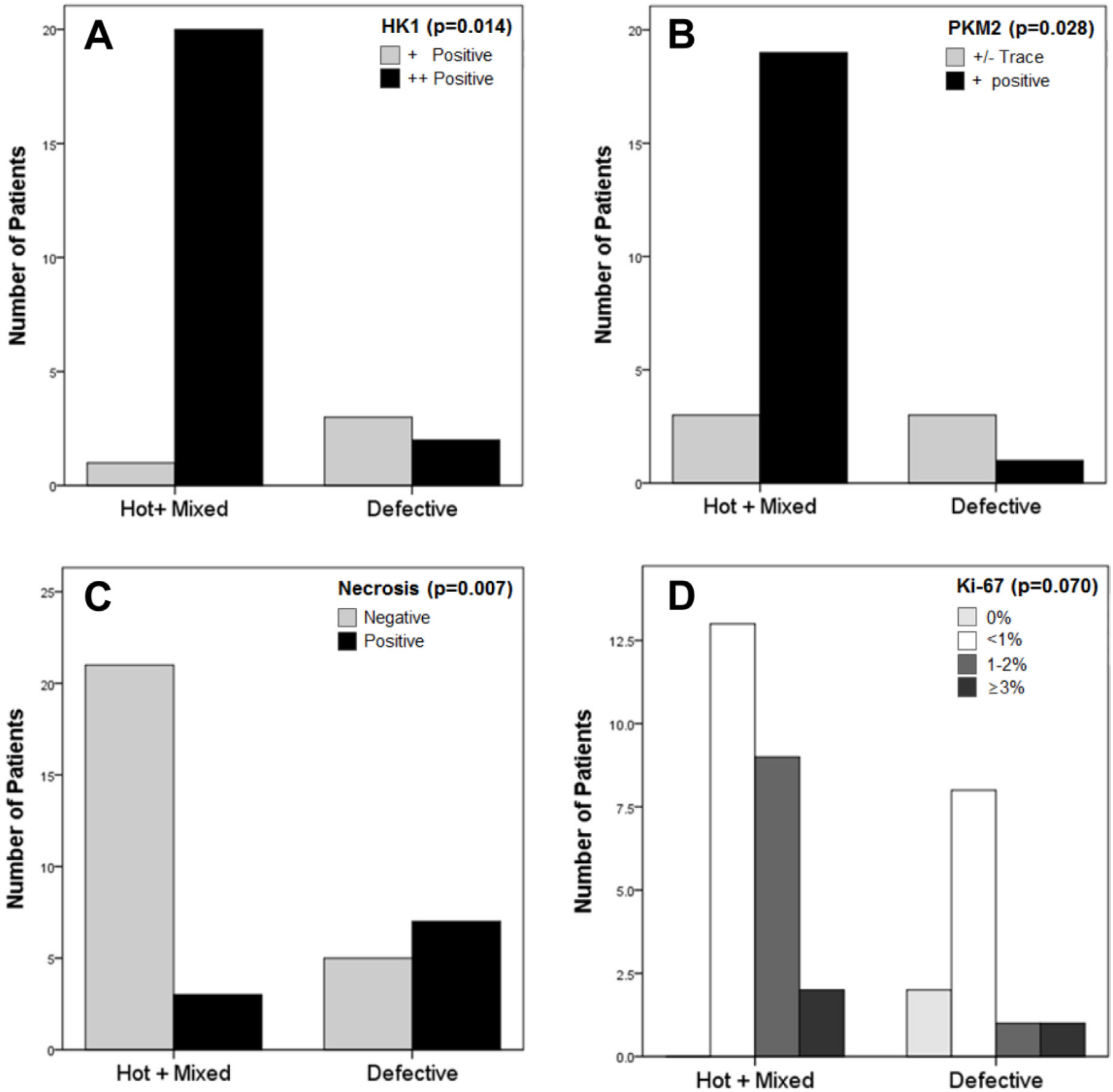

Supplementary Figure 3: Comparison between HK1, PKM2, Ki-67 expression and necrosis according to the pattern of ^{18}FDG-uptake. Expression of HK1 and PKM2 decreased in defective pattern, comparing with hot and mixed patterns (**A** and **B**). Intra-tumoral necrosis was significantly associated with defective type SPNs (**C**). Defective type had a tendency to show lower proliferation power, compared with hot and mixed SPNs (**D**).

Supplementary Table 1: Correlation between pattern of ^{18}FDG uptake and radiologic PET-parameters

Type	Proportion of 18FDG uptake	Age (years)	Radiologic Tumor Size (cm)	SUVmax (g/cm^3)	MTV2.5 (cm^3)
**Hot (*n* = 19)**	70% ≤	36.3 ± 10.8[a]	3.4 ± 2.2[a]	5.2 ± 2.8[a]	34.4 ± 69.9[a]
**Mixed (*n* = 5)**	30 ≤ < 70%	25.2 ± 13.9[a]	8.4 ± 3.6[b]	11.1 ± 7.1[b]	55.7 ± 64.4[a]
**Defective (*n* = 12)**	< 30%	33.4 ± 9.8[a]	5.6 ± 2.0[a]	3.6 ± 2.3[a]	16.8 ± 35.7[a]
p-value		0.146	0.002	0.001	0.462

[1]Statistical significances were tested by one-way analysis of variances (ANOVA) among groups. [2]The same letters (a, or b) in same column indicate non-significant differences between groups based on Turkey's multiple comparison test

Supplementary Table 2: Expression of glucose metabolism-related genes in SPN and PCA

Gene symbol	Gene ID	Gene description	Fold change (Tumor / Normal)			
			SPN	*p*-val	PCA	*p*-val
CTNNB1	3091	catenin (cadherin-associated protein), beta 1	4.38	0.003	1.70	0.398
SLC2A1 [GLUT1]	6513	solute carrier family 2 (facilitated glucose transporter), member 1	2.22	0.012	11.01	< 0.001
SLC2A6 [GLUT6]	11182	solute carrier family 2 (facilitated glucose transporter), member 6	2.47	0.031	2.12	0.035
SLC2A12 [GLUT12]	154091	solute carrier family 2 (facilitated glucose transporter), member 12,	19.82	< 0.001	2.13	0.067
SLC2A14 [GLUT14]	144195	solute carrier family 2 (facilitated glucose transporter), member 14	2.60	0.008	2.55	0.027
HK1	3098	hexokinase 1	7.01	< 0.001	3.72	0.006
HK2	3099	hexokinase 2	1.97	0.384	4.87	0.002
HK3	3101	hexokinase 3	-1.09	0.915	1.28	0.37
PFKM	5213	phosphofructokinase, muscle	5.35	< 0.001	3.68	0.001
ENO1	2023	Enolase 1	-1.12	0.921	2.61	< 0.001
ENO2	2026	Enolase 2	21.11	< 0.001	9.02	< 0.001
ENO3	2027	Enolase 3	1.56	0.085	1.34	0.372
PKM2	5315	pyruvate kinase, muscle	6.28	< 0.001	3.68	0.001
PDHB	5162	pyruvate dehydrogenase (lipoamide) beta	1.10	0.852	–1.14	0.370
LDHA	3939	lactate dehydrogenase A	–1.30	0.601	2.97	0.002

Supplementary Table 3: Immunohistochemical analysis of glucose metabolism-related genes in five PCA patients

#	GLUT1	HK1	ENO2	PKM2	PDH	LDHA	Ki-67
1	+	+	-	+++	+	++	30%
2	+	-	-	+++	+	++	8%
3	-	-	-	+++	+	++	20%
4	+	-	-	++	+	++	30%
5	+	-	-	++	+	++	30%

PCA; pancreatic ductal adenocarcinoma

Supplementary Table 4: Correlation between staining intensity of glucose metabolism-related genes and pattern of ^{18}FDG-uptake pattern in SPNs

		Clinical patterns of 18FDG-uptake		*P*-value
		Hot and Mixed	Defective	
**GLUT1**	-	4	1	0.646
	+	20	11	
**HK1**	+	1	3	0.014
	++	20	2	
**PKM2**	+/-	3	3	0.028
	+	19	1	
**Necrosis**	-	21	5	0.007
	+	3	7	
**Ki-67**	0	0	2	0.070
	< 1	13	8	
	1–2	9	1	
	3 ≤	2	1	
